# Exploring the effect of LncRNA DANCR to regulate the Keap1‐Nrf2/ARE pathway on oxidative stress in rheumatoid arthritis

**DOI:** 10.1002/iid3.1163

**Published:** 2024-01-24

**Authors:** Shaohong Cai, Yue Sun, Yuan Wang, Zhangying Lin

**Affiliations:** ^1^ Graduate School Anhui University of Traditional Chinese Medicine Hefei China; ^2^ Department of Rheumatology The First Affiliated Hospital of Anhui University of Traditional Chinese Medicine Hefei China

**Keywords:** LncRNA DANCR, oxidative stress, rheumatoid arthritis

## Abstract

**Introduction:**

Aberrant expression of long noncoding RNAs (LncRNAs) can regulate oxidative stress in rheumatoid arthritis (RA). This study focused on investigating the effects of LncRNA differentiation antagonizing nonprotein coding RNA (DANCR) regulation of Keap1‐Nrf2/ARE pathway on inflammation and oxidative stress in RA.

**Methods:**

The levels of LncRNA DANCR/miR‐486‐3p/Keap1 in peripheral blood of 30 RA groups and 30 normal subjects were examined, and the association of LncRNA DANCR with inflammatory indicators of RA was investigated. We stimulated fibroblast‐like synoviocytes (FLS) from RA patients with tumor necrosis factor α and subsequently performed in vitro cellular assays to construct overexpression plasmids and small interfering RNAs of LncRNA DANCR to investigate the relationship between LncRNA DANCR and FLSs viability and migration in RA, as well as the effects on cellular oxidative stress factors and Keap1‐Nrf2/ARE pathway; molecular biology analysis was used to predict microRNAs that can bind LncRNA DANCR, and luciferase verified the binding sites of LncRNA DANCR with Keap1 and miR‐486‐3p; to further refine the gene and protein expression results, we used reverse transcription‐quantitative polymerase chain reaction and immunoblotting assays.

**Results:**

In both groups of peripheral blood mononuclear cells, the expression levels of LncRNA DANCR and Keap1 messenger RNA were higher in the RA group than in the normal control group, and the opposite was true for miR‐486‐3p; LncRNA DANCR was positively correlated with total antioxidant capacity (TAOC), IL6, IL17, malondialdehyde (MDA), but not with IL11, rheumatoid factor, cyclic citrullinated peptide, superoxide dismutase (SOD), with 28‐joint disease activity score, reactive oxygen species, C‐reactive protein, and erythrocyte sedimentation rate were negatively correlated; overexpression of LncRNA DANCR stimulated the Keap1‐Nrf2/ARE pathway, decreased the expression of IL10, SOD, TAOC, and increased the expression levels of MDA, IL11, IL‐17, PD‐L1, and silencing of LncRNA DANCR was the opposite, but knockdown of miR‐486‐3p or overexpression of keap1 reversed the expression of the above‐mentioned inflammatory and oxidative factors. In addition, pcDNA‐DANCR clearly showed stronger cell invasion and migration ability and exacerbated its inflammatory response, which may be related to the regulatory role of miR‐486‐3p and Keap1‐Nrf2/ARE signaling pathway, and we verified their targeting relationship using dual luciferase, showing that DANCR could regulate Keap1‐Nrf2/ARE through miR‐486‐3p modulates the Keap1‐Nrf2/ARE pathway and affects inflammatory and oxidative responses in RA patients.

**Conclusion:**

The low‐expressed LncRNA DANCR may regulate the Keap1‐Nrf2/ARE pathway and suppress the inflammatory and oxidative responses in RA patients.

## Graphical abstract

Lowly expressed LncRNA DANCR ameliorates the immune inflammatory response in rheumatoid arthritis (RA) by sponging miR‐134‐5p to activate its mediated Keap1‐Nrf2/ARE signaling pathway, inhibiting the release of inflammatory factors from RA‐fibroblast‐like synoviocytes and stimulating antioxidant capacity

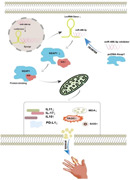

## 1 INTRODUCTION

Rheumatoid arthritis (RA) is a prevalent inflammatory autoimmune disorder distinguished by inflammation of the synovium and resultant joint damage.^1^ Activated fibroblastic synoviocytes (FLS), and inflammatory and oxidative factors are found to be involved in the inflammatory mechanisms of RA during the bone destruction and synovitis of RA.^2^ Oxidative stress generates excessive free radicals that oxidize other molecules in the body, for example, excessive free radicals in RA patients reduce the antioxidant level of the body and accelerate the progression of bone destruction.^3^ We demonstrated that the low expression of LINC00638 regulates the Nrf2/HO‐1 signaling pathway, triggering inflammation in RA and influencing its oxidative stress response.^4^ The Nrf2/HO‐1 signaling pathway can also affect antioxidant enzymes, regulate cellular redox responses, and influence other antioxidant enzymes.^5^ Proteins between Keap1 and Nrf2 can interact to affect oxidative‐antioxidant mechanisms, and when this interaction is inhibited, Nrf2 cannot be degraded, Keap1 is inhibited, and cytoplasmic Nrf2 concentrations increase and are transferred to the nucleus that accelerates the transcription of antioxidant‐related genes,^6^ so this article focuses on the upstream regulators of this pathway in RA.

Many LncRNAs also promote the development and progression of RA after dysregulated expression.^7^ Su et al. found that LncRNA HAND2‐AS1 sponged miR‐143‐3p/TNFAIP3 induced apoptosis and inhibited proliferation, invasion, migration, and inflammation of RA‐FLSs^8^; Sun et al. found that Lnc‐AL928768.3 promotes proliferation, invasion, and inflammation while inhibits apoptosis of RA‐FLS via activating lymphotoxin β‐mediated nuclear factor kappa B (NF‐κB) signaling.^9^ We had previously shown that the abnormally expressed LncRNA00638 has a regulatory role in RA. There are numerous studies demonstrating that LncRNA plays a role as a competitive endogenous RNA (ceRNA), competing with protein‐coding messenger RNA (mRNA) and binding to microRNA (miRNA) via sponge miRNA. Used bioinformatics Gene Ontology and Kyoto Encyclopedia of Genes and Genomes pathway to analyze DANCER‐targeted mRNAs and identified Nrf2 as the direct target gene of DANCER and Keap1‐Nrf2/ARE signaling pathway.^10^ To further predict the miRNAs related to and shared by DANCR and Nrf2, we obtained a mature sequence of related miRNAs according to reference PMID: 24151514. The target binding potential of DANCER and miRNAs was predicted by RNAhybRid, and a potential binding site with hsa‐miR‐486‐3p was identified. The potential binding site of keap1 to miR‐486‐3p was found by cross‐sectional analysis with bioinformatics software and database. Therefore, this study focuses on whether LncRNA DANCR can affect the oxidative stress effect in RA by regulating the Keap1‐Nrf2/ARE pathway.

## 2 DATA AND METHODS

### 2.1 General data

Thirty patients with RA, 13 males and 17 females, average age 45.72 ± 15.10 years, disease duration 1–23 years, average disease duration 8.70 ± 6.34 years, hospitalized in the Department of Rheumatology and Immunology, Anhui Provincial Hospital of Traditional Chinese Medicine were selected from May 2020 to May 2021; 30 cases of healthy control group were from the Health Examination Center of Anhui Provincial Hospital of Traditional Chinese Medicine, including 16 males and 14 females There was no statistically significant difference in the general data between the two groups. The study was approved by the ethics committee of this hospital, and both groups of subjects read and signed the informed consent form (ethics number 2019H‐12). This study complied with Strengthening the Reporting of Observational Studies in Epidemiology guidelines.

### 2.2 Diagnostic criteria

The studied RA patients all met the new classification criteria for RA jointly proposed by the American College of Rheumatology and the European League Against Rheumatism in 2010.^11^

### 2.3 Inclusion and exclusion criteria

Inclusion criteria: ① meeting the above diagnostic criteria and ② signing the informed consent form.

Exclusion criteria: ① not fulfilling the inclusion criteria, ② having serious diseases or severe extra‐articular manifestations, ③ having mental abnormalities, ④ being pregnant or lactating, and ⑤ being considered by the investigator as a contraindication.

### 2.4 Experimental instruments and equipment

OLYMPUS microscope; flow cytometer: Beckman‐Coulter XL‐ESPIC MCL flow cytometer.

Cell analyzer: Sysmex XT‐2000i, fully automatic cell analyzer; chemistry analyzer: HITACHI 7600, fully automatic chemistry analyzer; tabletop high‐speed freezer: model: Centrifuge 5417R, Eppendorf; electric thermostat: model: HH‐W21‐600, Shanghai Medical Technology Co. Ltd.; Plate washer: Model: ELX50, Bio‐TEK; Enzyme marker: Model: ELX800, Bio‐TEK.

High‐pressure steam sterilizer: Model: ES‐215, TOMY; Ultra‐pure water system: Model: AYJ1‐0501‐U, Aquapro; UV‐Vis spectrophotometer: Model: UV2401PC, SHIMADZU; RT‐PCR instrument: Daan Gene Co. Model: T1‐Thermoblock, T‐Gradient Thermoblock, Biometra; Electrophoresis instrument: Model: EPS‐301, Amersham; Gel image analyzer: Model: Gel Doc XR, Bio‐RAD.

### 2.5 Indicators of RA disease activity

After an early morning fast, 5 mL of intravenous lung fluids were drawn from the RA and normal control (NC) groups and placed in a tube for measurement of erythrocyte sedimentation rate (ESR), C‐reactive protein (CRP), rheumatoid factor (RF), and cyclic citrullinated peptide (CCP) using the Weil method. Disease is active when the DAS28 score is >2.6.

### 2.6 Experimental methods

#### 2.6.1 Cell culture and transfection

Tumor necrosis factor α (TNF‐α) was continuously stimulated in RA‐FLS cells for 24 h, and the cells were divided into six groups:

RA‐FLS, RA‐FLS+TNF‐α, RA‐FLS+TNF‐α+pcDNA‐NC, RA‐FLS+TNF‐α+pcDNA‐DANCR, RA‐FLS+TNF‐α+siRNA‐NC, and RA‐FLS+TNF‐α+siRNA‐DANCR.

#### 2.6.2 Detection of LncRNA DANCR/miR‐486‐3p/Keap1 expression in peripheral blood mononuclear cells (PBMCs) by reverse transcription‐quantitative polymerase chain reaction (RT‐qPCR)

RNA was extracted using Trizol, reverse‐transcribed, followed by amplification reaction and electrophoresis, and semi‐quantitative analysis using Gelpro32 gel image with β‐actin as internal reference, using Relative Quantification Study software and 2^−ΔΔ*C*^t analysis. The primers are shown in Table 1.

**Table 1:** Primer sequences.

Gene	Amplicon size (bp)	Forward primer (5′ → 3′)	Reverse primer (5′ → 3′)
β‐actin	96	CCCTGGAGAAGAGCTACGAG	GGAAGGAAGGCTGGAAGAGT
U6	94	CTCGCTTCGGCAGCACA	AACGCTTCACGAATTTGCGT
Keap1	193	ACTGTACCTGTTGAGGCACTTT	GCACATGATTCCCGCTTT
LncRNA DANCR	83	AGGTGGATTCTGTTAGGGA	AGTATTCAGGGTAAGGGTCA
has‐miR‐486‐3p		ACACTCCAGCTGGGCGGGGCAGCTCAGTA	TGGTGTCGTGGAGTCG
has‐miR‐486‐3p‐RT	CTCAACTGGTGTCGTGGAGTCGGCAATTCAGTTGAGATCCTG		

#### 2.6.3 Enzyme‐linked immunosorbent assay (ELISA) for cytokine levels

Elisa method was used to detect interleukin (IL)‐17, IL‐10, and IL‐11 levels.

#### 2.6.4 Biochemical method to measure superoxide dismutase (SOD), malondialdehyde (MDA), and total antioxidant capacity (TAOC)

The serum SOD was measured by chemiluminescence method, and the MDA and TAOC expression levels in serum or cell supernatant were detected by biochemical kits.

#### 2.6.5 Flow cytometry detection of reactive oxygen species (ROS)

Collect cells from each group mentioned above and adjust the cell concentration to (1–10) x 10^6^ cells/mL. Add a concentration of 10 μmol/L dichlorodihydrofluorescein diacetate (DCFH‐DA), incubate at 37°C for 20 min, mix every 3–5 min to ensure thorough interaction between the probe and cells. Set cells without staining as negative controls. Wash the cells twice with phosphate‐buffered saline to remove DCFH‐DA that has not entered the cells. First, calibrate the voltage using negative control cells. Adjust the voltage at the forward scatter‐side scatter light position, encircle the desired cell cluster in the histogram, and further adjust the voltage to ensure that the negative control's *x*‐axis position is to the left of 10^4^. After voltage calibration, individually analyze each sample from different groups using flow cytometry.

#### 2.6.6 Cell counting kit 8 method to detect cell viability

Take the RA‐FLS at logarithmic growth stage to make cell suspension and measure the absorbance value after incubation.

#### 2.6.7 Cell migration assay (Transwell)

Trypsin digestion of log phase RA‐FLS, centrifuged and collected after termination, made into cell suspension, cultured without serum, and removed for fixation and staining. Observe the cell invasion number after rinsing.

#### 2.6.8 Detection of protein expression by Western blot analysis

Collect cell samples, add radioimmunoprecipitation assay cell lysate for lysis, and collect total protein. Refer to the relevant instructions and use the electrochemiluminescence kit to detect the proteins. See Study 1 for the specific sequence and method of operation.

#### 2.6.9 Dual luciferase assay to verify the binding site of LncRNA DANCR with Keap1 and miR‐486‐3p

The miR‐486‐3p mimic or its NC was cotransfected into RA‐FLS with LncRNA DANCR‐WT, LncRNA DANCR‐MUT (mutant), Keap1‐WT (wild type) and Keap1‐MUT vectors, and luciferase activity was measured.

### 2.7 Statistical methods

SPSS 25.0 statistical software was used for analysis. The data of each group were expressed as mean ± standard error (±), *t* test was used for comparison between two groups, one‐way analysis of variance was used for comparison between multiple groups, GraphPad 9.4.1 was used for graphing, and *p* < .05 indicated that the differences were statistically significant.

## 3 RESULTS

### 3.1 Clinical data of the study subjects

ESR, CRP, RF, and anti‐CCP were elevated in RA patients compared with healthy controls (**p < .01), with statistically significant differences; the mean DAS28 score in the RA group was 5.02 ± 0.82 (Table 2).

**Table 2:** General clinical information.

**Indicators (mean ± SD)**	**NC (*n*** = **30)**	**RA (*n*** = **30)**
ESR (mm/h)	7.77 ± 3.01	59.03 ± 25.27**
CRP (mg/L)	1.98 ± 1.21	29.72 ± 14.55**
RF (U/mL)	5.32 ± 2.21	131.21 ± 96.04**
Anti‐CCP (U/mL)	2.45 ± 1.36	161.09 ± 41.18**
DAS28	—	5.02 ± 0.82

Abbreviations: Anti‐CCP, anti‐cyclic citrullinated peptide antibody; CRP, C‐reactive protein; DAS28: 28‐joint disease activity score; ESR, erythrocyte sedimentation rate; NC, normal control; RA, rheumatoid arthritis; RF, rheumatoid factor; SD, standard deviation

** *p* < 0.01.

### 3.2 LncRNA DANCR/miR‐486‐3p/Keap1 gene expression and serum levels of oxidative factors in clinical subjects

**Figure 1:**
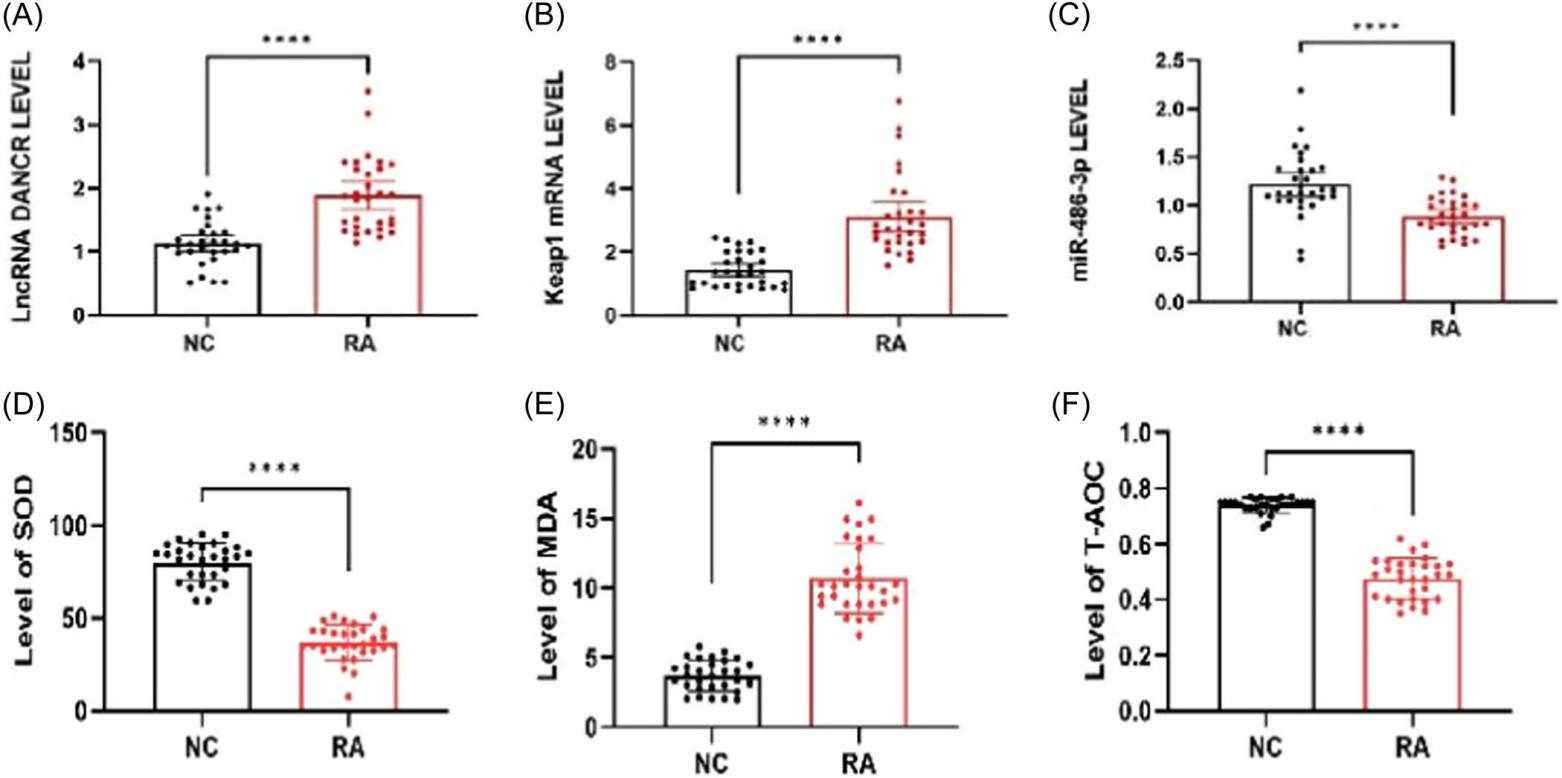
LncRNA DANCR/miR‐486‐3p/Keap1 gene expression levels and serum oxidation factor levels. (There are 30 cases in both the RA and NC groups, and a *t* test was conducted.) (A)–(C) Expression of LncRNA DANCR, miR‐486‐3p, Keap1 mRNA. (D) and (E) Serum levels of SOD (superoxide dismutase), MDA (malondialdehyde), and TAOC (total antioxidant capacity).

The results showed that LncRNA DANCR and Keap1 mRNA expression levels were significantly higher and miR‐486‐3p mRNA expression levels were significantly lower in the RA group (*p* < .0001) (Figure 1A–C). Serum MDA levels were significantly higher and SOD and total antioxidant capacity (TAOC) levels were significantly lower in RA patients (Figure 1D–F).

### 3.3 Correlation analysis of LncRNA DANCR levels with inflammation and oxidative stress in RA patients

LncRNA DANCR was positively correlated with IL17, RF, IL6, SOD, anti‐CCP, MDA, and TAOC and negatively correlated with age, disease duration, DAS28, ESR, CRP, IL11, and ROS (Table 3).

**Table 3:** 

**Indicators**	***r***	***p* Value**
Age	−.03	.523
Duration of disease	−.42	.426
DAS28	−.25	.013
ESR	−.31	.017
CRP	−.11	.032
RF	.2	.297
Anti‐CCP	.22	.245
IL‐6	.31	.023
IL‐11	−.06	.721
IL‐17	.16	.032
ROS	−.09	.047
SOD	.04	.852
MDA	.23	.017
TAOC	.26	.014

Abbreviations: Anti‐CCP, anti‐cyclic citrullinated peptide antibody; CRP, C‐reactive protein; DAS28, 28‐joint disease activity score; ESR, erythrocyte sedimentation rate; IL‐6, IL‐17, and IL‐11, interleukins; MDA, malondialdehyde; RF, rheumatoid factor; ROS, reactive oxygen species; SOD, superoxide dismutase; TAOC, total antioxidant capacity.

### 3.4 Effect of LncRNA DANCR on FLS invasion, migration, and cell viability

**Figure 2:**
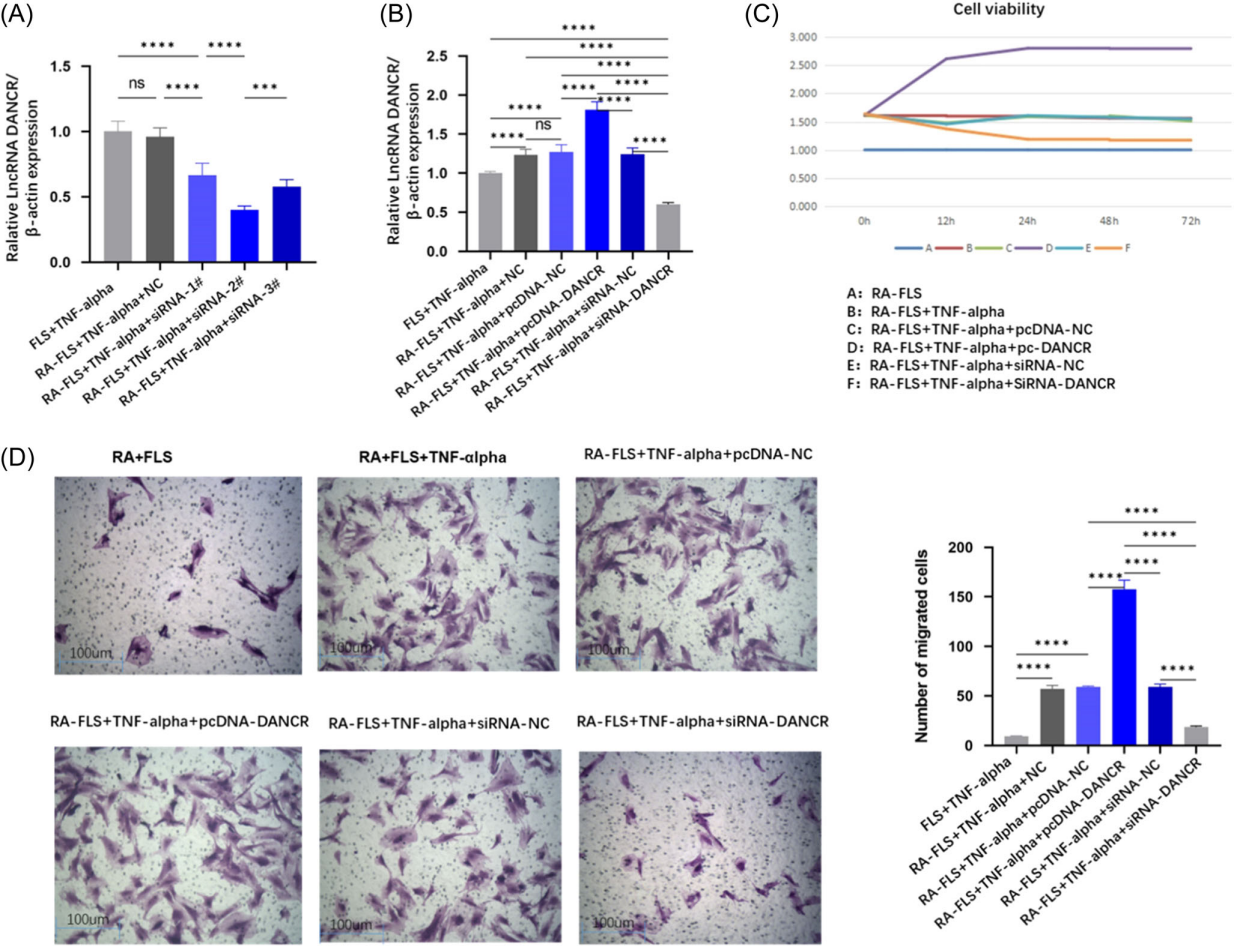
Effect of LncRNA DANCR on FLS migration and cell viability. (Each group has four samples, and a parametric test was conducted.) (A) Screening of optimal siRNA in TNF‐α transfected FLS cells; (B) LncRNA DANCR expression in TNF‐α stimulated RA‐FLS transfected with overexpression plasmid and siRNA. (C) Assay of LncRNA DANCR by cell counting kit 8 assay on LncRNA DANCR TNF‐α‐stimulated RA‐FLS proliferation. (D) Effect of LncRNA DANCR on RA‐FLS migration under TNF‐α stimulation was assessed by Transwell assay (scale bar, 100 μm). FLS, fibroblast‐like synoviocytes; NC, normal control; pcDNA, plasmid cloning DNA; RA, rheumatoid arthritis; RF, rheumatoid factor; siRNA, small interfering RNA; TNF‐α, tumor necrosis factor α.

The expression level of transfected LncRNA DANCR in si‐RNA2# was low after TNF‐α stimulation (Figure 2A), and we subsequently transfected LncRNA DANCR overexpression plasmid and small interfering RNA (siRNA) in RA‐FLS (Figure 2B). We examined the proliferation and migration of RA‐FLS to investigate the effect of LncRNA‐DANCR on TNF‐α stimulated RA‐FLS. siRNA‐DANCR clearly showed weaker proliferation and migration ability (Figure 2B–D).

### 3.5 Effect of LncRNA DANCR on oxidative stress indicators

**Figure 3:**
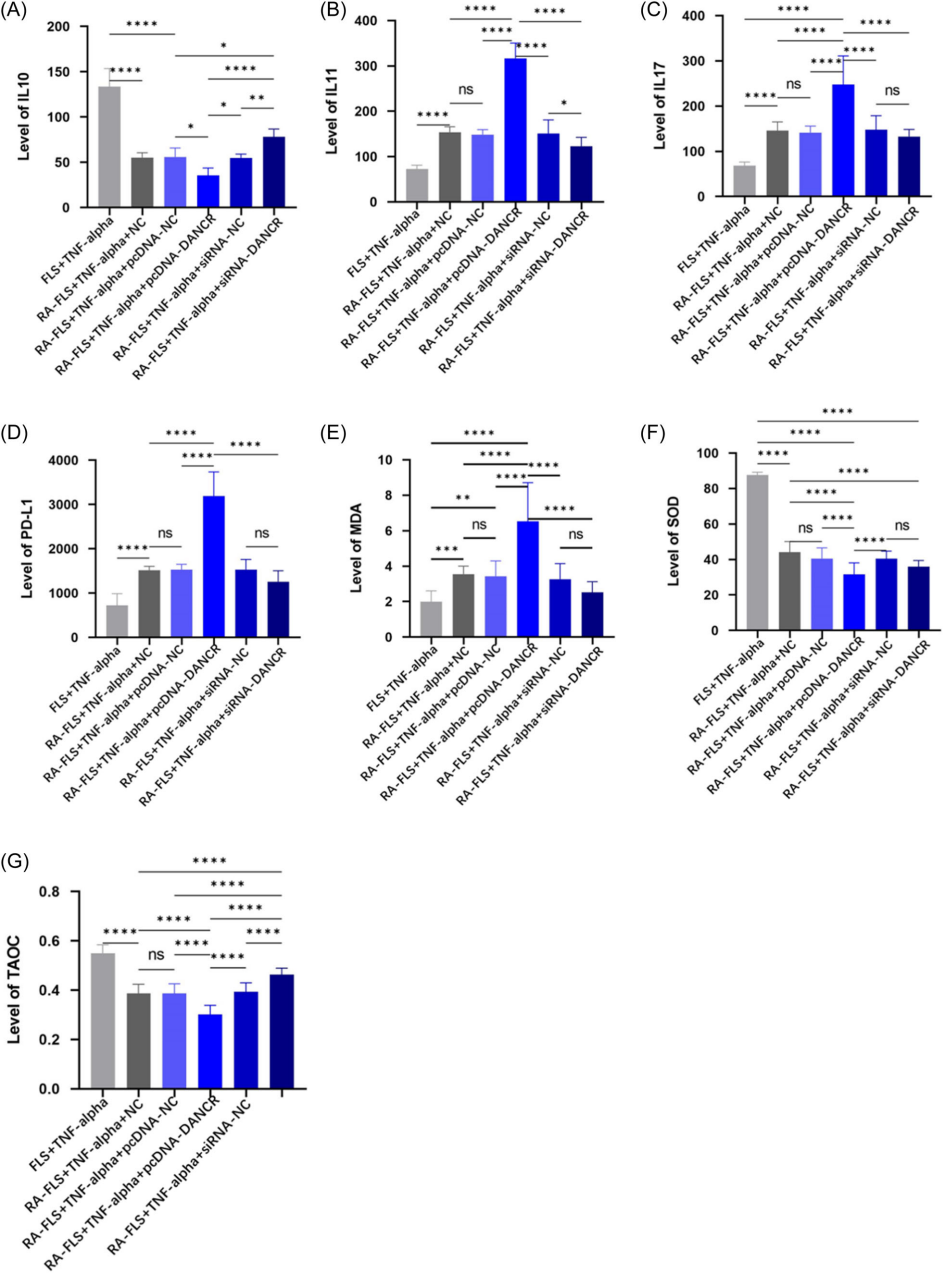
Effects of LncRNA DANCR on oxidative stress indicators. (Each group has six samples, and a parametric test was conducted.) (A)–(D) Enzyme‐linked immunosorbent assay to detect the expression of IL‐10, IL‐11, IL‐17, and PD‐L1. (E)–(G) Biochemical method to determine the levels of MDA, SOD, and TAOC. FLS, fibroblast‐like synoviocytes; IL‐10, IL‐11, and IL‐19, interleukins; MDA, malondialdehyde; NC, normal control; pcDNA, plasmid cloning DNA; RA, rheumatoid arthritis; RF, rheumatoid factor; siRNA, small interfering RNA; SOD, superoxide dismutase; TAOC, total antioxidant capacity; TNF‐α, tumor necrosis factor α.

To observe the inflammatory and oxidative stress effects of LncRNA DANCR on TNF‐α‐stimulated FLS cells, we examined the expression of IL10, IL11, IL17, and PD‐L1. We found that low expression of LncRNA DANCR increased the level of IL10 and inhibited the expression of IL11, IL‐17, and PD‐L1 (Figure 3A–D). We observed MDA, TAOC, and SOD levels by biochemical method and found that transfection of siRNA‐DANCR increased the expression of SOD and TAOC and decreased the level of MDA (Figure 3E–G)

### 3.6 LncRNA DANCR regulates miR‐486‐3p/Keap1 gene expression level in TNF‐α stimulated RA‐FLS

**Figure 4:**
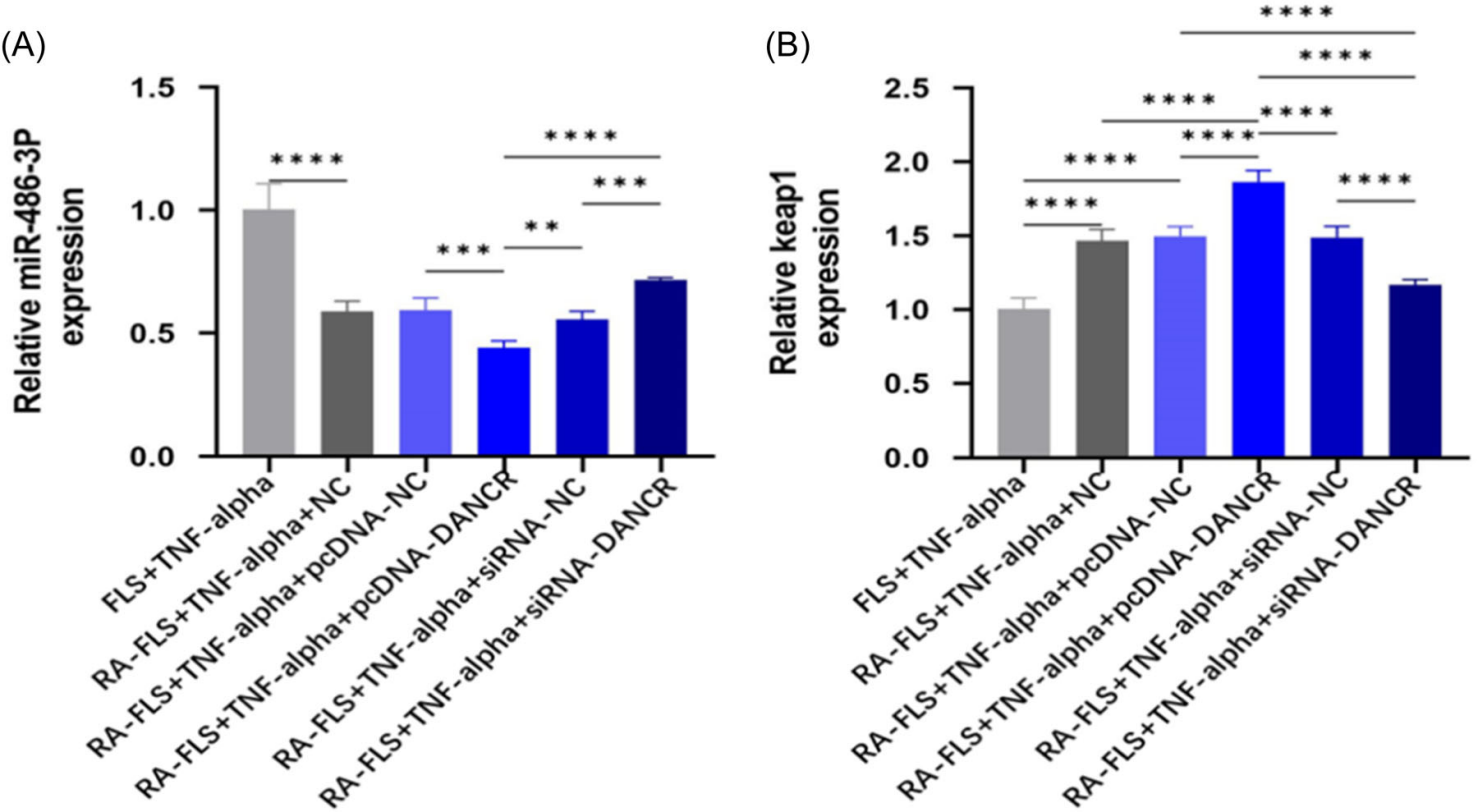
LncRNA DANCR regulates miR‐486‐3p/Keap1 gene expression levels. (Each group has six samples, and a parametric test was conducted.) (A) miR‐486‐3p gene expression levels in TNF‐α‐stimulated RA‐FLS by q‐PCR. (B) Keap1 in TNF‐α‐stimulated RA‐FLS gene expression levels. FLS, fibroblast‐like synoviocytes; NC, normal control; pcDNA, plasmid cloning DNA; RA, rheumatoid arthritis; TNF‐α, tumor necrosis factor α.

The level of miR‐486‐3p was elevated in the SiRNA‐DANCR group (Figure 4A) and Keap1 expression was decreased (Figure 4B). The opposite was true in the plasmid cloning DNA (pcDNA)‐DANCR group (Figure 4). The results indicated that low expression of LncRNA‐DANCR negatively regulated keap1, while positively regulating miR‐486‐3p.

### 3.7 Effect of knocking down LncRNA DANCR, silencing miR‐486‐3p, and overexpressing keap1 on inflammatory response and oxidative stress

**Figure 5:**
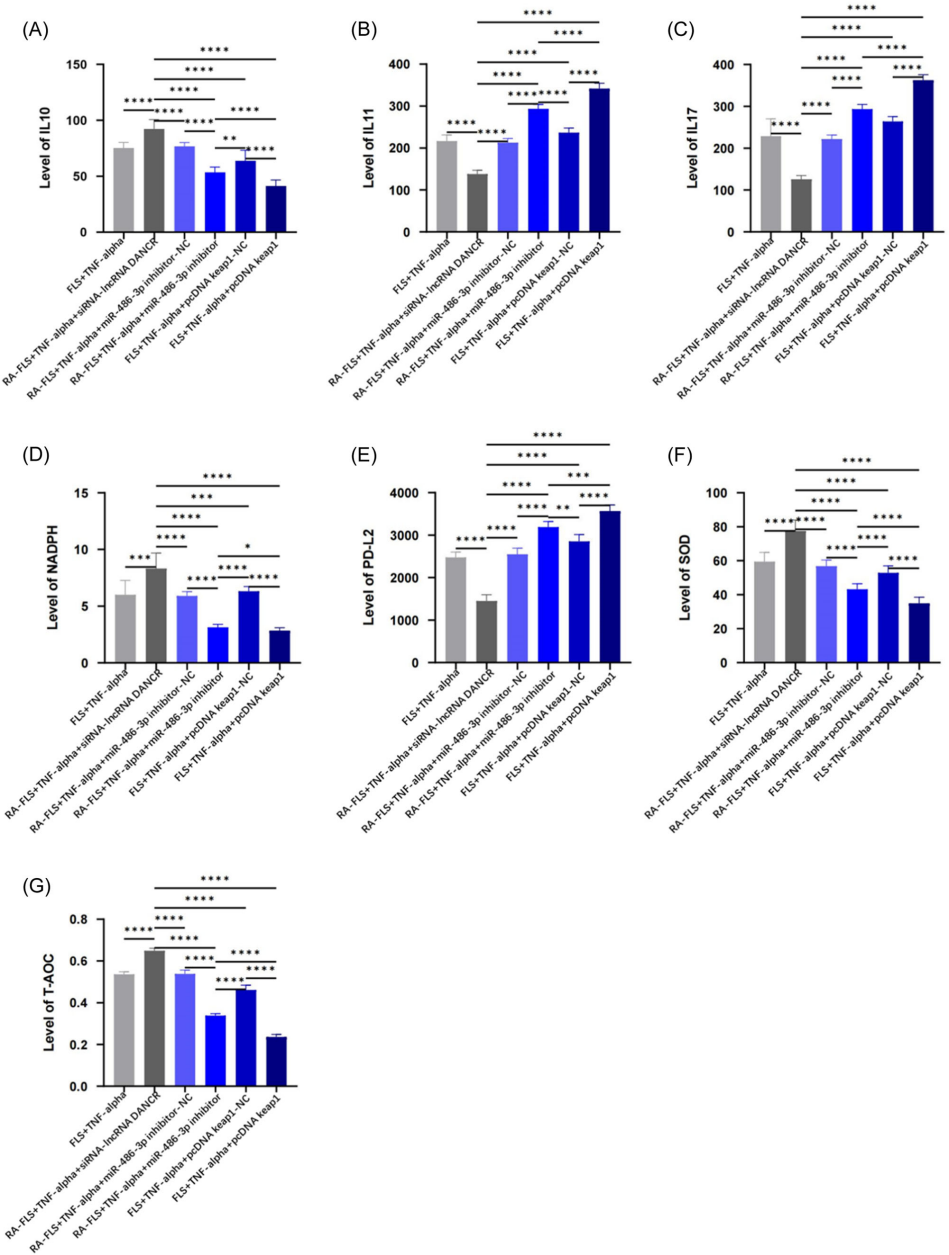
Effect of knockdown of LncRNA DANCR, silencing miR‐486‐3p, and overexpression of keap1 on inflammatory response and oxidative stress. (Each group has six samples, and a parametric test was conducted.) (A)–(D) Enzyme‐linked immunosorbent assay for IL‐10, IL11, IL‐17, and PD‐L2 expression. (E)–(G) Biochemical assay for MDA, SOD, and PD‐L2, monoclonal antibody; NADPH, reduced nicotinamide adenine dinucleotide phosphate. FLS, fibroblast‐like synoviocytes; IL‐10, IL‐11, and IL‐19, interleukins; MDA, malondialdehyde; NC, normal control; pcDNA, plasmid cloning DNA; RA, rheumatoid arthritis; siRNA, small interfering RNA; SOD, superoxide dismutase; TAOC, total antioxidant capacity; TNF‐α, tumor necrosis factor α.

When we knocked down the LncRNA DANCR gene, the expression of IL10, NADPH, SOD, and TAOC factors was stimulated, while the levels of IL11, IL17, and PDL‐2 were reduced. The results of silencing miR‐486‐3p and overexpressing keap1 gene were opposite (Figure 5).

### 3.8 Changes in protein expression levels of Keap1 and Nrf2 in RA patients

**Figure 6:**
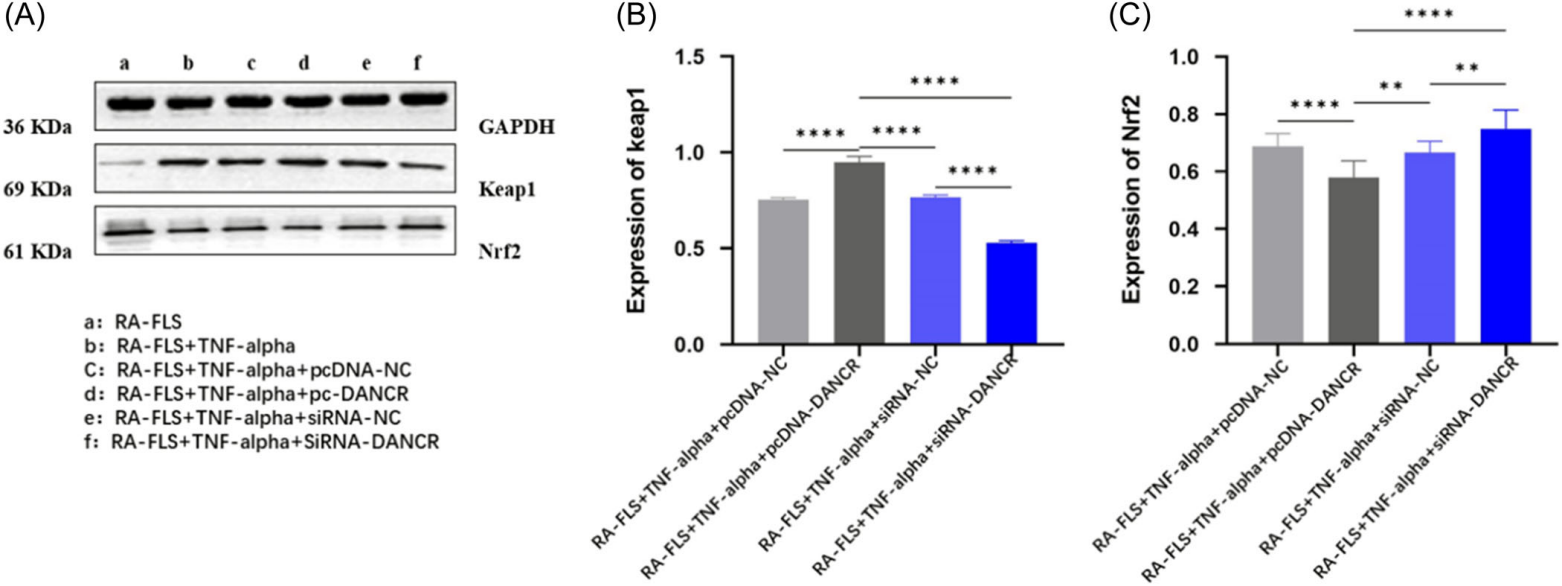
Changes in protein expression levels of Keap1 and Nrf2 in RA‐FLS. (Each group has six samples, and a parametric test was conducted.) (A)–(C) Protein levels of Keap and Nrf2 gene expression in RA‐FLS, respectively. FLS, fibroblast‐like synoviocytes; NC, normal control; pcDNA, plasmid cloning DNA; RA, rheumatoid arthritis; siRNA, small interfering RNA; SOD, superoxide dismutase; TNF‐α, tumor necrosis factor α.

To investigate whether LncRNA DANCR activates and regulates the Keap1‐Nrf2 pathway, analysis was performed using Western blot, and we found that transfection of pcDNA‐LncRNA DANCR promoted the level of keap1 and suppressed the expression of Nrf2, while the opposite result was obtained after silencing LncRNA DANCR (Figure 6).

### 3.9 Dual luciferase validation of the binding site between LncRNA DANCR and miR‐486‐3p, Keap1

**Figure 7:**
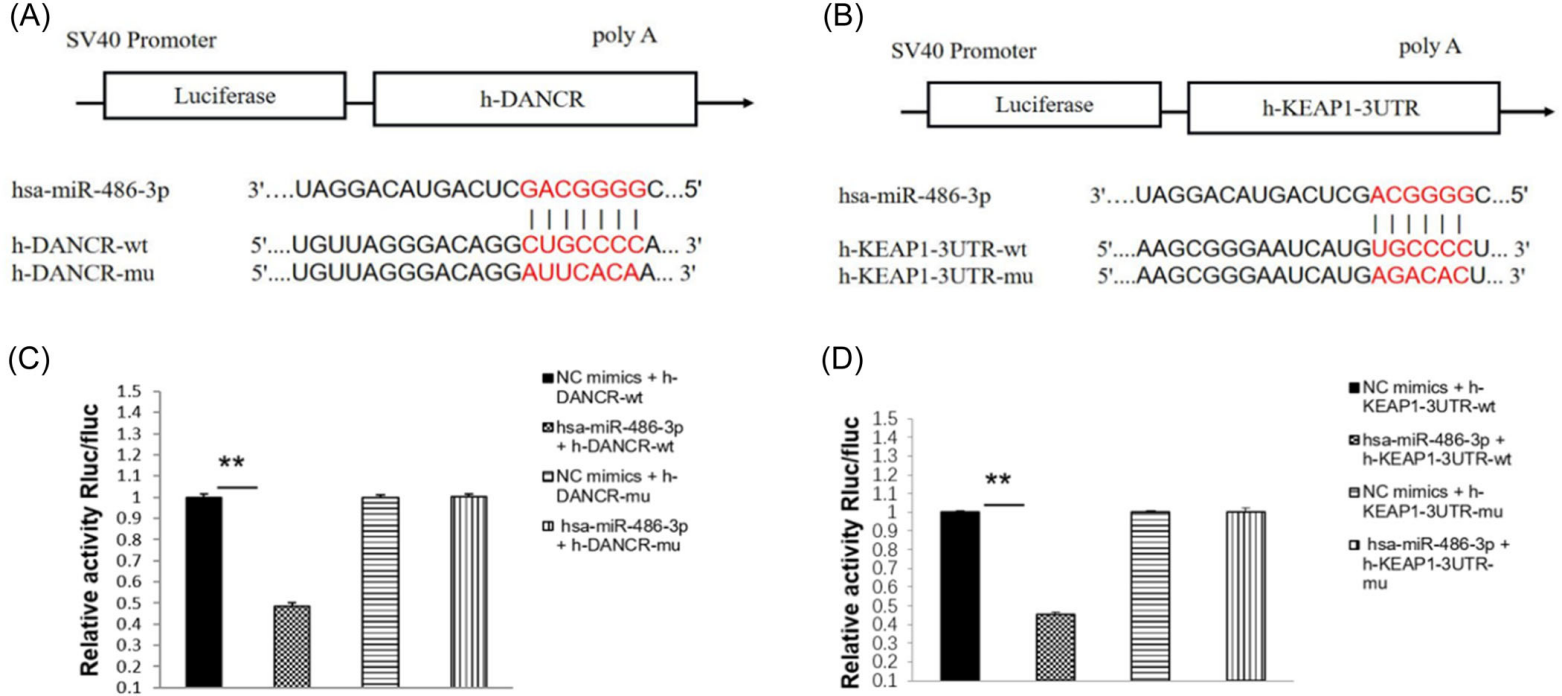
Dual luciferase validation of the binding sites between LncRNA DANCR and miR‐486‐3p, Keap1. (A) and (B) miR‐486‐3p binding sites of LncRNA DANCR and Keap1 3′UTR region. (C) Experimental results of LncRNA DANCR with miR‐486‐3p. (D) miR‐486‐3p and Keap1 experimental results.

The gene template and mutation template of LncRNA binding sequence 3′UTR and the gene template and mutation template of Keap1 binding sequence 3′UTR were inserted into the gene vector to form the expression plasmid (Figure 7A,B). After the addition of miR‐486‐3p mimics, the LncRNA DANCR‐WT luciferase activity was reduced, while the MUT luciferase activity was not significantly different from the control enzyme activity, and there was direct target binding of LncRNA DANCR to miR‐486‐3p (Figure 7C). The WT luciferase activity of Keap1 decreased after the addition of miR‐486‐3p mimics, indicating that Keap1 has a direct binding site with miR‐486‐3p (Figure 7D).

## 4 DISCUSSION

As our knowledge of the transcriptome space expands, it is becoming increasingly clear that LncRNAs have multiple microRNA recognition binding sites as competing endogenous RNAs (ceRNAs) or natural microRNA sponges that can eliminate the endogenous repression of their target transcripts by miRNAs. For example, LncRNA‐EWSAT1 can induce apoptosis and inhibit proliferation by affecting RA‐FLS through downregulation of miR‐326 levels,^12^ LncRNA XIST binding to GATA1 leads to upregulation of CCN6, which causes RA to enter the inflammatory response phase by altering synovial fibroblast proliferation and angiogenic activity,^13^ and LncRNA HOTAIR affects FLSsl growth and apoptosis by regulating miR‐106b‐5p/Smad signaling and thus.^14^ These reports suggest that these aberrantly expressed LncRNAs regulate the inflammatory and oxidative stress effects of RA. We found in this study that LncRNA DANCR levels were higher in the RA group and that LncRNA DANCR levels were positively correlated with serum inflammatory and oxidative factor levels in RA patients.

To further investigate whether LncRNA DANCR affects oxidative stress and inflammatory factors in RA, we measured oxidative and inflammatory factors in RA‐PBMC using biochemical assays (Figure 1), and we hypothesized that LINCRNA DANCR modulates the antioxidant and inflammatory responses in RA.

We all know that oxidative stress is associated with inflammation and both factors are closely linked to RA, oxidative stress affects immune cell metabolic processes and promotes inflammation in RA,^15^ Therefore, some traditional medications operate through a new mechanism of inhibiting oxidative stress and increasing antioxidants to alleviate clinical symptoms in patients with RA.^16^ The results of our study demonstrated elevated MDA expression and significantly decreased SOD and TAOC expression in the serum of RA patients, suggesting an imbalance of oxidative homeostasis and immune inflammatory response in RA patients. In addition, we did Spearman's correlation analysis (Table 3), which demonstrated that the aberrantly expressed LncRNA DANCR has the potential to regulate RA inflammatory and oxidative responses.

FLS proliferation contributes to the pathogenesis of RA and accelerates joint destruction, and it has been shown that FLS cells in RA resemble tumor‐like cells that actively enhance their invasive proliferation and migration and can produce inflammatory‐like changes, for example, LncRNA MIR31HG regulates the inflammatory response in RA‐FLS, inhibits the proliferation and invasive capacity of cells associated with RA, and increases the level of inflammation (Table 3) and makes inflammation levels elevated^17^; downregulation of LncRNA XIST regulates miR‐126‐3p/NF‐κB and inhibits FLS cells proliferation, thereby suppressing RA disease activity.^18^ We found that low expression of LncRNA DANCR inhibited RA‐FLS proliferation and migration (Figure 2).

LncRNAs can also regulate other different inflammatory and oxidative stress processes. LncRNA TUG1 regulates miR‐34b‐5p/GAB1 to reverse lung inflammation and apoptosis; LncRNA CYLD‐AS1 binds miR‐134‐5p and affects oxidative stress and inflammatory factor expression in RPE cells.^19^ We found that silencing LncRNA DANCR decreased MDA, IL11, IL‐17, PD‐L1 levels and increased IL10, SOD, and TAOC levels.^20^ Therefore, silencing LINCRNA DANCR inhibits the inflammatory response of RA‐FLS and thus improves its antioxidant capacity.

**Figure 8:**
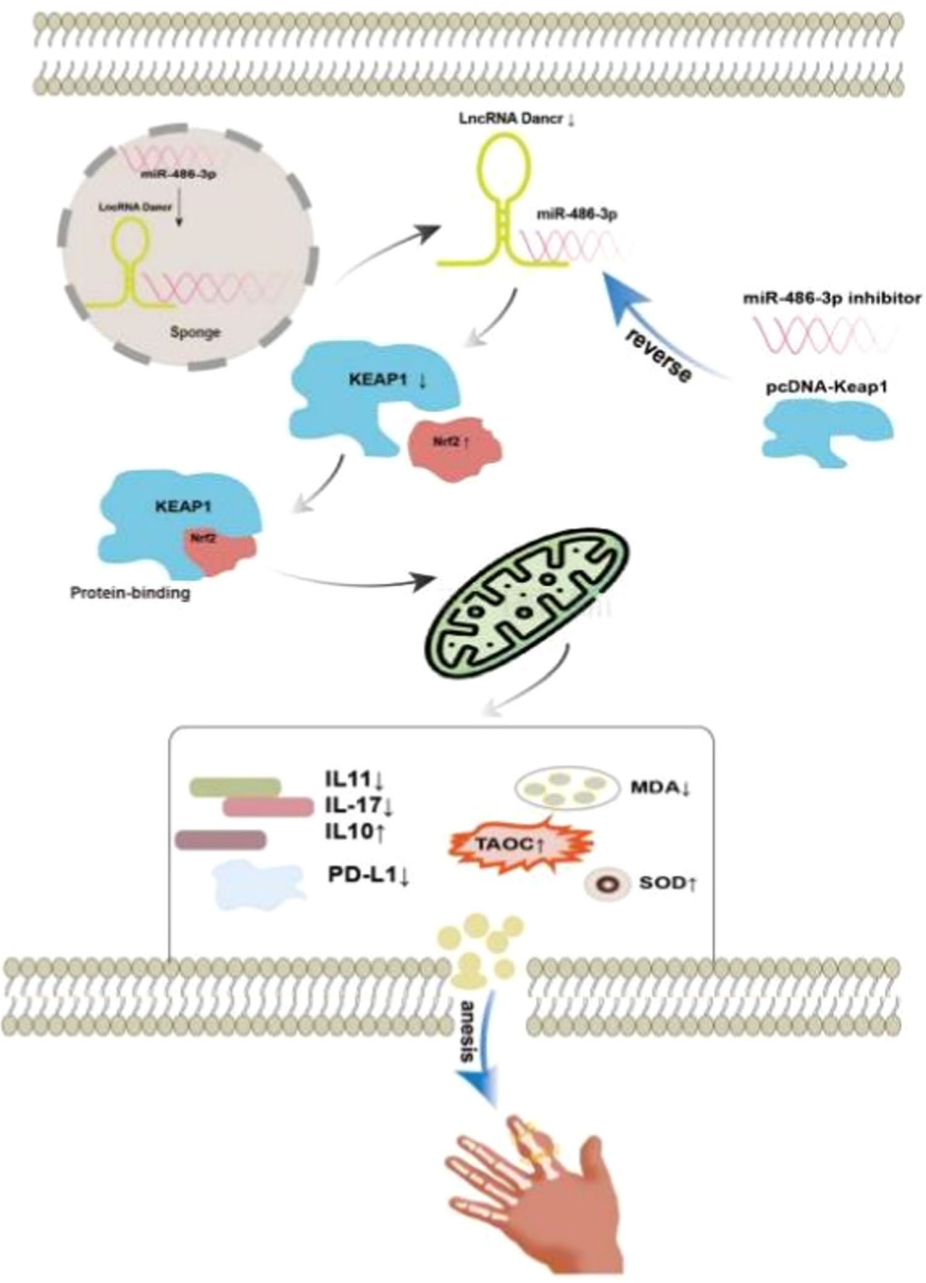
Illustration of the mechanism: downregulation of LncRNA DANCR regulates the Keap1‐Nrf2/ARE signaling pathway in combination with miR‐134‐5p, inhibits inflammatory factor release, enhances antioxidant capacity, and improves RA immune inflammatory response. Overexpression of keap1 and knockdown of miR‐134‐5p reversed these results.

The Keap1/Nrf2/ARE signaling pathway can be involved in maintaining cellular homeostasis under conditions of stress and inflammation, carcinogenesis, and apoptosis.^21^ Data from Li et al. demonstrated that PX can inhibit the level of oxidative stress and reverse aristolochic acid‐induced renal insufficiency through the Keap1‐Nrf2/HO‐1 signaling pathway, Nrf2 protein interactions inhibit Keap1 and regulate various genes including HO‐1.^22^ In this study, RT‐qPCR results in PBMCs of subjects showed that LncRNA DANCR, Keap1 mRNA expression levels were significantly elevated and miR‐486‐3p mRNA expression levels were significantly decreased in the RA group. After transfection, we found that low expression of LncRNA‐DANCR negatively regulated keap1 and positively regulated miR‐486‐ We showed by WB data that transfection of siRNA‐DANCR not only regulated keap1 but also promoted Nrf2 expression. These results suggest the existence of LncRNA DANCR/miR‐486‐3p/keap1 functional axis and activation of Keap1‐Nrf2/ARE pathway. And then we found that silencing miR‐486‐3p and overexpressing keap1 gene could reverse the effect of knockdown of LncRNA DANCR on inflammation and oxidative response, and we verified the existence of direct target binding of LncRNA DANCR, Keap1, and miR‐486‐3p. Thus, we propose that lowly expressed LncRNA DANCR ameliorates the immune inflammatory response in RA by sponging miR‐134‐5p to activate its mediated Keap1‐Nrf2/ARE signaling pathway, inhibiting the release of inflammatory factors from RA‐FLS and stimulating antioxidant capacity (Figure 8). Sample size in this study is relatively small, which might limit our ability to generalize to the overall population; larger‐scale studies could help confirm the universality of the effects observed.

## AUTHOR CONTRIBUTIONS

Shaohong Cai performed the data analysis and collection and writing. Yuan Wang supervised and revised the article. Yue Sun researched and designed the experiments. Zhangying Lin participated in organizing the experimental data. All authors read and approved the final manuscript.
